# Immunosenescence and inflammaging in Parkinson’s disease: mechanisms and therapeutic prospects

**DOI:** 10.3389/fimmu.2026.1749278

**Published:** 2026-02-12

**Authors:** Ruyu Yan, Hongfang Feng, Jie Zhang

**Affiliations:** 1 Queen Mary School, Jiangxi Medical College, Nanchang University, Nanchang, Jiangxi, China; 2 The First Affiliated Hospital, College of Clinical Medicine, Henan University of Science and Technology, Luoyang, Henan, China; 3 Department of Neurology, The First Affiliated Hospital, Jiangxi Medical College, Nanchang University, Nanchang, Jiangxi, China

**Keywords:** immunosenescence, neuroinflammation, Parkinson’s disease, senescence-associated secretory phenotype, therapeutic strategies

## Abstract

Parkinson’s disease (PD) is characterized by the progressive loss of dopaminergic neurons, with growing evidence underscoring the critical role of immunosenescence—the age-related dysregulation of the immune system—in its pathogenesis. This review delineates the intricate interplay between systemic immunosenescence, chronic neuroinflammation, and neurodegeneration in PD. We explore how age-related remodeling of the peripheral immune system, termed “inflammaging,” promotes a pro-inflammatory milieu that compromises blood-brain barrier integrity and drives microglial activation within the central nervous system. A central focus is the senescence-associated secretory phenotype, a cocktail of pro-inflammatory factors released by senescent glial cells, which perpetuates a self-sustaining cycle of neuroinflammation, facilitates the propagation of pathological α-synuclein, and ultimately accelerates neuronal loss. The review further examines the disruption of vital neuroimmune communication pathways, including aberrant neuron-glia and gut-brain axis signaling, which are corrupted in the aging brain. We evaluate the translational promise of emerging therapeutic strategies designed to target this immunosenescence-neuroinflammation axis. These include senolytic agents to clear senescent cells, adoptive regulatory T-cell therapy, cytokine-targeted immunomodulation, and immune rejuvenation approaches. Finally, we discuss significant translational challenges and outline future research directions, emphasizing the need for advanced model systems, biomarker development, and AI-driven personalized medicine to successfully develop disease-modifying immunotherapies that disrupt the vicious cycle of immunosenescence and neurodegeneration in PD.

## 1 Introduction

Parkinson’s disease (PD) stands as the second most common neurodegenerative disorder, characterized by the progressive loss of dopaminergic neurons in the substantia nigra pars compacta. This neuronal degeneration is responsible for the classic motor symptoms of bradykinesia, rigidity, and tremor, as well as a spectrum of non-motor manifestations. While the pathological hallmark of PD is the presence of Lewy bodies containing aggregated α-synuclein, the precise drivers of disease progression remain incompletely understood. The etiology of PD involves a complex interplay of genetic susceptibility (e.g., mutations in LRRK2and GBA), environmental exposures, and lifestyle factors, with advanced age representing the predominant risk factor.

In PD, senescence can be broadly categorized into two interconnected types: primary senescence and secondary senescence. Primary senescence is largely driven by intrinsic aging processes, such as replicative exhaustion due to telomere attrition and the accumulation of genomic instability over time. This form of senescence contributes to a baseline susceptibility by globally impairing cellular function and homeostasis. Conversely, secondary senescence is induced by various pathological stressors. Chronic neuroinflammation, characterized by the persistent presence of pro-inflammatory cytokines, is a predominant instigator of this stress-induced state. Furthermore, pathological factors central to PD, including oxidative stress and the aggregation of α-synuclein, act as potent inducers of cellular senescence. The relevance of this distinction for PD pathogenesis lies in their convergence on a common destructive pathway: the senescence-associated secretory phenotype (SASP). Whether initiated by age-related exhaustion or inflammatory and proteotoxic stress, senescent cells release a potent mix of SASP factors. This creates a vicious, self-reinforcing cycle wherein the SASP from primarily senescent cells exacerbates local inflammation, which in turn drives secondary senescence in neighboring cells. This cycle amplifies neuroinflammation, impairs the clearance of pathological proteins, and ultimately accelerates dopaminergic neurodegeneration. Central to this dysregulation is the chronic activation of microglia, the resident immune cells of the central nervous system (CNS). With advancing age, microglia transition into a primed, pro-inflammatory state, persistently releasing cytotoxic mediators such as TNF-α and IL-1β, which contribute to neuronal injury and exacerbate disease pathology. Clinical observations support this link, demonstrating correlations among microglial activation, elevated cerebrospinal fluid inflammatory markers, and PD severity (1).

This central neuroinflammation is critically shaped by systemic immunosenescence-the age-related functional decline of the peripheral immune system. Immunosenescence is characterized by a weakened adaptive immune response, including a contracted naïve T-cell repertoire and the accumulation of dysfunctional memory T cells. This systemic inflammatory milieu can exacerbate neuroinflammation within the CNS, establishing a self-reinforcing cycle that accelerates nigrostriatal degeneration (2). Consequently, the bidirectional communication between a senescent peripheral immune system and a primed CNS environment is now considered a critical axis in PD pathophysiology.

Given the central role of immune dysregulation, therapeutic strategies aimed at modulating the immune response represent a promising strategy in PD research. Emerging interventions, such as enhancing regulatory T-cell function, inhibiting specific pro-inflammatory signaling pathways (e.g., TNF-α or IL-1β pathways), and adopting lifestyle modifications like physical exercise, have demonstrated potential in dampening neuroinflammation in both preclinical models and early clinical settings (3). Such approaches aim to break the cycle of chronic inflammation and modify the course of the disease.

In this review, we critically examine the intricate interplay between immunosenescence, chronic neuroinflammation, and neurodegeneration in PD e. We explore the core mechanisms underpinning this relationship, including microglial senescence, the senescence-associated secretory phenotype (SASP), and disruptions in intercellular communication within the neuroimmune axis. Furthermore, the review evaluates the translational promise of emerging immunomodulatory strategies, with a particular focus on advancing disease-modifying therapies that target the critical interface between aging and the neuroimmune system.

## 2 The interface of systemic and CNS immunosenescence in PD

### 2.1 Systemic immunosenescence and inflammaging

**Figure 1:**
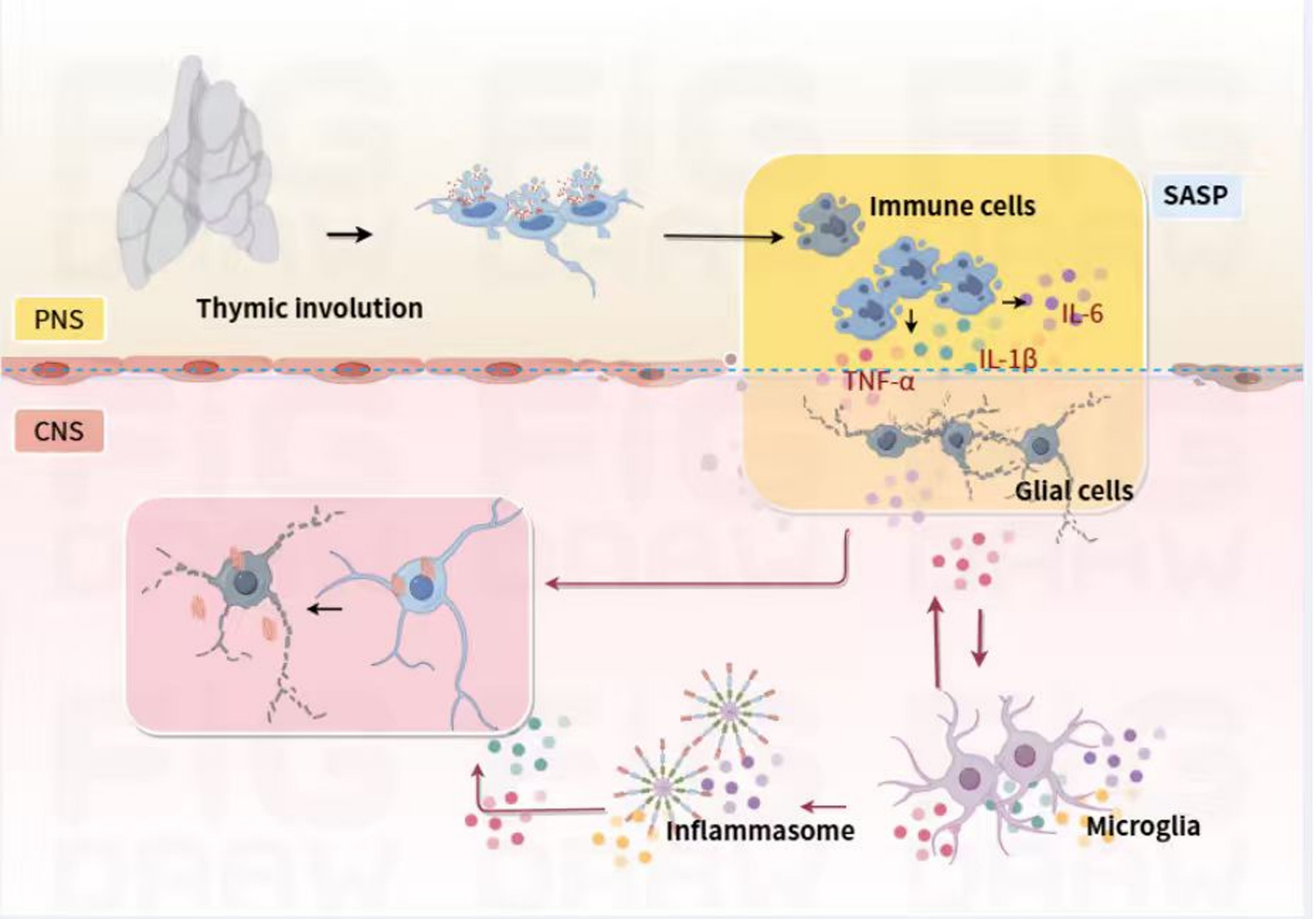
Schematic diagram illustrating the peripheral-central immune interaction mechanism in PD. 1) Systemic immunosenescence is defined by thymic involution, accumulation of senescent memory T cells, and defective innate immune cell function, which drive inflammaging—a state of chronic low-grade systemic inflammation mediated by the SASP. 2) Within the CNS, immunosenescence is marked by glial cell dysfunction and compromised BBB integrity. Senescent glial cells secrete pro-inflammatory SASP factors, and increased BBB permeability permits infiltration of peripheral inflammatory cytokines; these events together initiate neuroinflammaging. The synergistic and additive actions of systemic immunosenescence and local CNS inflammation induce sustained microglial activation (including NLRP3 inflammasome hyperactivation), promote pathogenic protein aggregation, and ultimately cause dopaminergic neuron loss in PD.

Systemic immunosenescence refers to the progressive, age-related decline in immune function across the organism. Key characteristics include thymic involution—resulting in reduced output of naïve T cells and a contracted T-cell receptor repertoire—as well as the accumulation of senescent, terminally differentiated memory T cells (e.g., CD28^−^/CD57^+^ subsets) (see **Figure 1**). These adaptive immune alterations are accompanied by innate immune dysfunction, such as diminished phagocytic capacity and impaired antigen presentation by macrophages and dendritic cells. Clinically, immunosenescence manifests as reduced vaccine efficacy, increased susceptibility to infections (4, 5). This state arises from the immune dysregulation described above and contributes to the systemic inflammatory milieu.

### 2.2 CNS immunosenescence and neuroinflammaging

Within the aging central nervous system, immunosenescence manifests as progressive dysfunction of glial cells and the blood-brain barrier (BBB). Microglia contribute to neuronal damage (6). Concurrently, astrocytes show signs of senescence and diminished homeostatic support, further impairing neuronal resilience (7). These maladaptive glial alterations disrupt metabolic regulation, synaptic integrity, and tissue repair, collectively fostering a chronically inflamed CNS environment.

The BBB also undergoes age-related deterioration, with structural impairments-including degradation of tight junction proteins (e.g., Claudin-5) and the endothelial glycocalyx-increasing its permeabilityy (8, 9). This allows peripheral immune cells and inflammatory mediators from the systemic inflammaging pool to infiltrate the CNS (10). Neuroinflammaging thus emerges from the confluence of systemic immunosenescence, BBB dysfunction, and glial senescence. This milieu, defined by persistent microglial activity and infiltration of peripheral immune cells, significantly exacerbates dopaminergic neuron loss in PD (11, 12). Despite growing preclinical and clinical evidence supporting these mechanisms, critical aspects remain incompletely elucidated. The temporal and causal relationships between systemic immunosenescence and CNS-specific inflammation require validation in longitudinal human studies. Furthermore, the heavy reliance on rodent models, which incompletely recapitulate human aging or sporadic PD, represents a significant translational gap. Therefore, future research must prioritize clarifying these underlying mechanisms in human contexts and validating therapeutic interventions that target the immunosenescence-neuroinflammaging axis to slow or prevent PD progression.

### 2.3 Neuroimmune crosstalk: peripheral mediators of central inflammation

Peripheral immunosenescence significantly influences PD pathogenesis by disrupting neuroimmune communication. Age-related shifts in the adaptive immune system-such as the contraction of the naïve T-cell repertoire and the expansion of antigen-experienced memory T cells-foster a pro-inflammatory milieu (“inflammaging”) that facilitates immune cell trafficking into the CNS (see **Figure 1**). This infiltration is further promoted by BBB dysfunction, as evidenced by elevated levels of activated T cells and monocytes in the cerebrospinal fluid of PD patients (13). Some viruses (e.g., SARS-CoV-2, HSV-1, and Cytomegalovirus (CMV)) and bacteria can initiate a systemic inflammatory response. For instance, SARS-CoV-2 infection may be associated with an increased risk of neurodegenerative sequelae, potentially by exacerbating underlying neuroinflammatory pathways (14). Furthermore, we cite preclinical and clinical data linking lifelong CMV exposure to an accelerated immune risk profile characterized by an increased proportion of terminally differentiated effector memory T cells, which is indicative of immunosenescence and is associated with chronic inflammation (15). This involves the release of pro-inflammatory cytokines (e.g., IL-1β, IL-6, TNF-α) into the bloodstream, which can compromise the integrity of the blood-brain barrier (BBB). A more permeable BBB allows these inflammatory mediators to enter the central nervous system and can facilitate the infiltration of peripheral immune cells, thereby priming or exacerbating neuroinflammation. After the acute infection is controlled, the initial immune activation can leave a lasting imprint on the immune system. More critically, latent viruses can periodically reactivate, especially in the context of an aging immune system. Each reactivation event places a repeated antigenic stress on the immune system, contributing to immunosenescence by promoting the clonal expansion of memory T cells (e.g., CD8+ CD28- T cells) and reducing the diversity of the T-cell repertoire, a hallmark of an aging immune system. This state of chronic, low-grade inflammation (“inflammaging”) creates a pro-inflammatory milieu that can accelerate neurodegeneration. Communication between peripheral inflammation and the CNS occurs via both humoral pathways (e.g., cytokine transport across circumventricular organs or the compromised BBB) and neural routes (e.g., afferent signaling via the vagus nerve) (16, 17).

Upon entering the CNS, peripheral immune cells and inflammatory mediators activate resident glia, particularly microglia and astrocytes (**Figure 1**). Infiltrating cells exacerbate BBB damage through the secretion of matrix metalloproteinases (MMPs), reinforcing a vicious cycle of neuroinflammation (18). Perturbations of the gut–brain axis further amplify this dynamic. Intestinal dysbiosis and increased permeability lead to elevated levels of pro-inflammatory cytokines and bacterial products like LPS. These mediators can stimulate afferent vagal nerve fibers by binding to receptors (e.g., TLR4) on the nerve terminals embedded in the gut mucosa (19, 20). The signal travels to the brainstem’s nucleus of the solitary tract (NTS) and is relayed to higher brain regions, ultimately promoting microglial activation in vulnerable areas like the SNpc, thereby “priming” central neuroinflammation. Notably, human epidemiological studies and vagotomy data suggest that interrupting this neural pathway may reduce the risk of PD development, underscoring its clinical relevance.

Systemic “inflammaging” compromises BBB integrity through downregulation of tight junction proteins and increased endothelial adhesion molecule expression (21). The resulting inflammatory microenvironment within the CNS promotes α-synuclein (αSyn) pathology and creates a self-sustaining feedback loop that drives neurodegeneration. Age-related dysbiosis increases intestinal permeability, allowing bacterial products to enter the systemic circulation. This can prime peripheral immune responses and may lead to the generation of αSyn-reactive T-cells, potentially initiating autoimmune reactions that contribute to early PD pathogenesis. This highlights how peripheral immunosenescence can influence the CNS through multiple interconnected pathways. Crucially, the detrimental effects of this neuroimmune crosstalk are not merely due to the presence of infiltrating cells or systemic factors, but are fundamentally mediated by their sustained, pathological signaling.

### 2.4 Core pathogenic mechanisms: impaired immune surveillance and α-synuclein

Age-related immune decline impairs the clearance of pathological α-synuclein (αSyn), promoting Lewy body formation, a hallmark of PD (22). Senescent microglia exhibit reduced phagocytic ability, hindering αSyn removal. Preclinical studies show that αSyn preformed fibrils (PFFs) induce heightened neuroinflammation and T-cell infiltration in aged mice, accelerating neuronal loss (3). As illustrated in **Figure 1**, aggregated αSyn activates microglial pattern recognition receptors (TLRs) and the NLRP3 inflammasome, initiating pro-inflammatory cascades (4, 22). This creates a vicious cycle: neuroinforcement encourages further αSyn misfolding and aggregation, which in turn intensifies inflammation. Additionally, aging disrupts proteostasis mechanisms, such as autophagy-lysosomal and ubiquitin-proteasome systems, leading to intracellular accumulation of misfolded αSyn (11).

## 3 SASP: a central driver of neuroinflammation

The SASP comprises a diverse spectrum of secreted bioactive molecules that function as critical paracrine and endocrine signals. This secretory profile establishes a chronically inflamed tissue microenvironment and significantly contributes to age-related pathology. While heterogeneous, SASP components can be broadly classified into: (1) Pro-inflammatory Cytokines: Such as Interleukin-6 (IL-6), IL-1β, and Tumor Necrosis Factor-alpha (TNF-α), which are primary drivers of neuroinflammation; (2)Chemokines: Such as CCL2 (MCP-1) and CXCL8 (IL-8), which are responsible for recruiting immune cells (e.g., neutrophils, monocytes) to sites of inflammation; (3) Growth Factors and Angiogenic Factors: Such as Vascular Endothelial Growth Factor (VEGF), which can compromise blood-brain barrier (BBB) integrity (23); (4) Proteases: Such as Matrix Metalloproteinases (MMPs) (24–26). The SASP is not static; its composition and intensity are shaped by the initiating stressor, cell type, and local microenvironment, underpinning its complex and context-dependent role in neurodegeneration.

### 3.1 Key upstream regulatory pathways of the SASP

**Figure 2:**
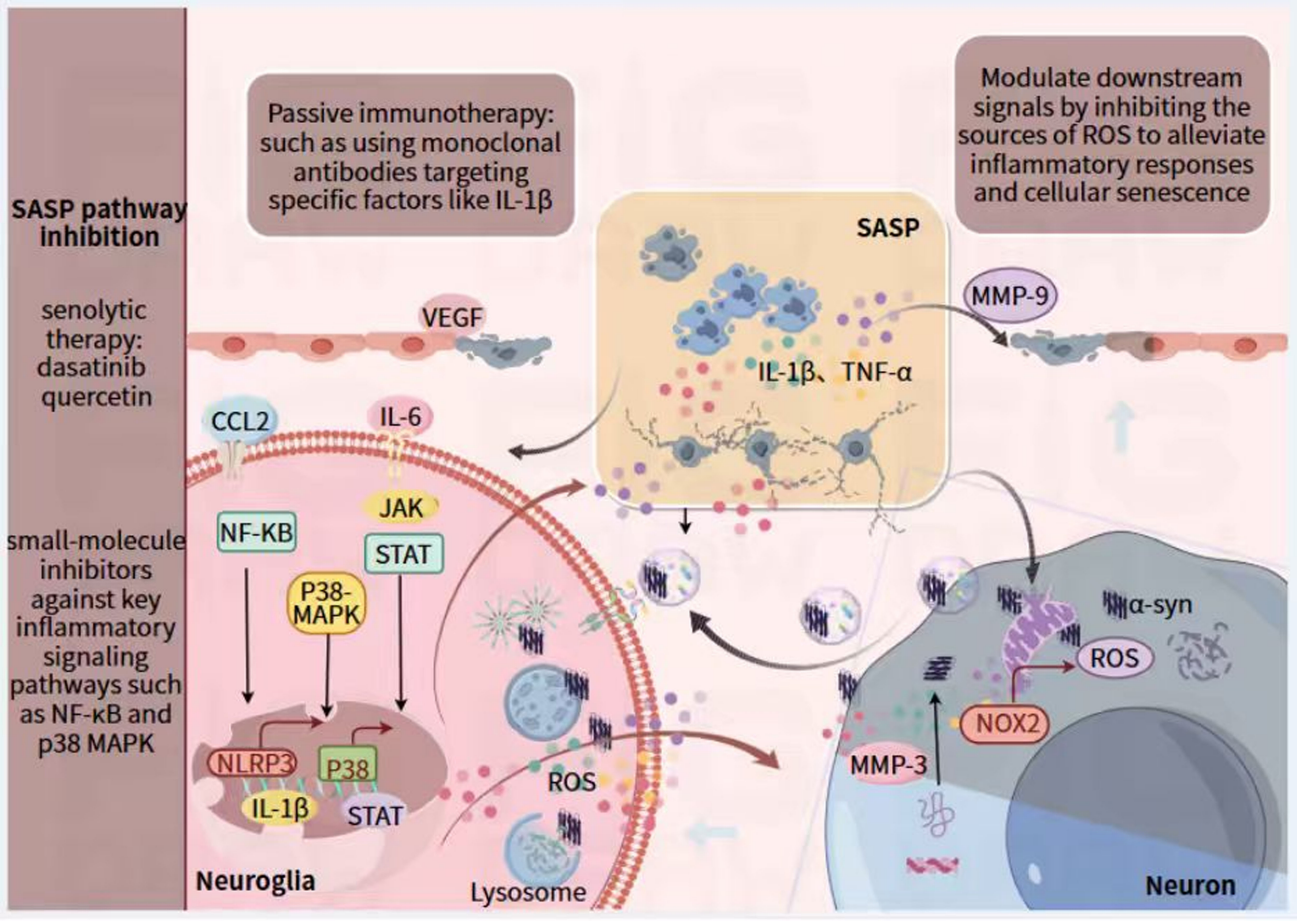
Therapeutic strategies targeting the inflammaging and SASP cycle in PD. 1) SASP-related factors disrupt the BBB through matrix metalloproteinase-mediated degradation of tight junction proteins, promote the infiltration of peripheral immune cells, and induce the aggregation, misfolding, and propagation of α-synuclein. Meanwhile, these factors can activate microglia/astrocytes into a pro-inflammatory phenotype, triggering the release of cytotoxic mediators and DAMPs. DAMPs further induce secondary SASP production in glial cells, forming a vicious cycle of neuroinflammation and dopaminergic neuron loss. Oxidative stress (e.g., NADPH oxidase 2-derived ROS) amplifies this cycle, thereby exacerbating cellular senescence. 2) Therapeutic strategies targeting SASP include: senolytics that selectively eliminate senescent glial cells (dasatinib in combination with quercetin), SASP neutralization therapy (anti-interleukin-1β monoclonal antibodies), and inhibitors of oxidative stress/signaling pathways (inhibitors of NADPH oxidase 2, NF-κB signaling, and p38 MAPK).

As illustrated in **Figure 2**, the dynamic regulation of SASP is governed by extrinsic factors—such as the nature of cellular stress, cell type, and tissue context—through core signaling cascades, primarily NF-κB and p38 MAPK, which operate synergistically to amplify and sustain the SASP. The NF-κB pathway serves as the principal transcriptional regulator of SASP components. It is activated during senescence by persistent DNA damage response (DDR) or other stressors, leading to its nuclear translocation and the induction of multiple pro-inflammatory cytokines and chemokines, including IL-6 and IL-8 (27). This process is further amplified by the cGAS–STING pathway, which senses cytoplasmic chromatin fragments and strongly potentiates NF-κB activation (28). Concurrently, the p38 MAPK pathway acts synergistically with NF-κB, both by enhancing its transcriptional activity and by stabilizing SASP factor mRNAs, thereby augmenting protein production and prolonging inflammatory signaling (27). These pathways, through positive feedback loops and interactions with epigenetic mechanisms—such as histone modification changes—convert transient stress into a persistent, self-reinforcing SASP, perpetuating chronic neuroinflammation.

### 3.2 Core pathological roles of the SASP in the central nervous system

The SASP components collectively compromise blood-brain barrier integrity, where VEGF directly impairs endothelial function while matrix metalloproteinases including MMP-3 and MMP-9 proteolytically degrade tight junction proteins such as Claudin-5 and Occludin, resulting in increased barrier permeability (27), which is presented in **Figure 2**. This structural compromise facilitates the infiltration of peripheral inflammatory cells and mediators into the CNS parenchyma, establishing a self-reinforcing cycle of “inside-out and outside-in” inflammation.

Concurrently, SASP factors including TNF-α, IL-1β and CCL2 operate through paracrine signaling to maintain surrounding microglia in a persistently activated M1-like state, characterized by NLRP3 inflammasome hyperactivation and excessive production of reactive oxygen species, ultimately leading to the release of additional neurotoxic substances that exacerbate neuronal damage (27). Additionally, the SASP enhances intercellular transmission of pathological α-synuclein via extracellular vesicles such as exosomes, thereby accelerating the spread of pathology throughout vulnerable neural circuits.

### 3.3 The self-reinforcing cycle of SASP in PD pathogenesis

The dysfunctional neuroimmune crosstalk in PD fosters a pro-inflammatory central nervous system (CNS) environment, which is perpetuated primarily by the SASP. This malignant feedback loop tightly couples chronic neuroinflammation with progressive neurodegeneration. Key SASP components, including IL-6 and TNF-α, exacerbate α-synuclein aggregation and misfolding, directly promoting Lewy body formation. For instance, IL-6 activates the JAK-STAT pathway, thereby accelerating α-synucleinopathy (29, 30). Moreover, SASP factors drive microglial and astrocytic polarization toward pro-inflammatory phenotypes (e.g., M1 microglia and A1 astrocytes), resulting in sustained release of cytotoxic mediators such as reactive oxygen species (ROS) and IL-1β, which inflict damage on dopaminergic neurons in the substantia nigra (**Figure 2**). A critical amplifier of this cycle is the release of damage-associated molecular patterns (DAMPs)-including HMGB1 and mitochondrial DNA (mtDNA)-from injured neurons. These DAMPs activate microglial Toll-like receptors (TLRs) and inflammasomes, intensifying neuroinflammation and inducing a secondary SASP in neighboring glial cells. This feed-forward loop not only sustains chronic inflammation but also propagates neurodegeneration (31, 32).

### 3.4 Targeting the neuron-glia interface: senolytics and SASP inhibition in PD

The pathological interplay between neurons and glial cells initiates a self-amplifying inflammatory cascade that critically contributes to disease progression in Parkinson’s disease. This detrimental cycle is frequently triggered by neuronal injury or the accumulation of pathological proteins such as α-synuclein aggregates, which function as DAMPs, which activate surrounding microglia and astrocytes, driving them toward a SASP (33). The resulting SASP-mediated neuroinflammation, characterized by the sustained release of pro-inflammatory factors, in turn inflicts secondary damage on vulnerable neurons, thereby reinforcing and perpetuating the pathological loop (34, 35).

The degenerative cycle is further exacerbated by the presence of misfolded protein aggregates. Specifically, α-synuclein preformed fibrils (PFFs) have been demonstrated to directly induce cellular senescence in both astrocytes and microglia, as evidenced by a decline in lamin B1 and HMGB1, coupled with an increase in p21 expression-well-characterized biomarkers of cellular senescence (36). Oxidative stress also plays a critical role in this process. The generation of reactive oxygen species (ROS), largely via NOX2 activation, promotes lipid peroxidation and mitochondrial dysfunction (37). These processes can stabilize p53 and activate the p38 MAPK pathway, thereby further propagating cellular senescenc (38, 39). Significantly, SASP can further establish a self-reinforcing circuit that markedly accelerates neuronal loss.

To disrupt the deleterious cycle linking neuroinflammation and neurodegeneration in PD, several therapeutic strategies targeting distinct nodes of this cascade are under investigation. Among these, senolytic drugs are designed to selectively clear senescent glial cells. Preclinical studies, including those utilizing mouse models injected with α-synuclein PFFs, have demonstrated that eliminating these cells can attenuate neuroinflammation and confer protection to dopaminergic neurons (40). Concurrently, SASP neutralization strategies aim to directly block the detrimental effects of the senescent cell secretome without eliminating the cells themselves (41, 42). Furthermore, suppressing oxidative stress and its downstream signaling by inhibiting ROS sources (e.g., using NOX2 inhibitors) or modulating key pathways (e.g., HMGB1-TLR4 or p38 MAPK) is also considered a viable approach to mitigate inflammation and cellular senescence (35, 43). Collectively, these approaches constitute a multi-target intervention strategy, offering promise for the development of disease-modifying therapies for PD.

### 3.5 SASP and the self-perpetuating cycle of neurodegeneration

Preclinical studies in PD models have demonstrated that inhibition of the SASP effectively reduces neuroinflammation, attenuates the loss of dopaminergic neurons, and improves motor function (44). Complementing this approach, senolytic agents, including dasatinib and quercetin, which selectively clear senescent cells, have been shown to mitigate SASP-mediated damage and promote functional recovery (45). The rationale for employing senolytics is particularly strong in PD, given the documented accumulation of senescent cells that actively contribute to disease progression. By eliminating these cells, senolytic therapies attenuate the pro-inflammatory SASP microenvironment and thereby support mechanisms of neural recovery. Current research efforts are consequently prioritized toward developing novel senolytic compounds with enhanced blood-brain barrier (BBB) permeability, representing a promising therapeutic paradigm for improving treatment efficacy in PD and other central nervous system disorders (46). The therapeutic potential of targeting the SASP is further supported by observations indicating a subsequent elevation in neurotrophic factors (e.g., BDNF, GDNF) and a reduction in oxidative stress following intervention (47). However, the pleiotropic roles of certain SASP components raise legitimate concerns about potential on-target side effects.

## 4 The impact of immunosenescence on intercellular communication in PD

The age-related decline in immune competence not only impairs the protective functions of immune cells but also actively fuels a cycle of chronic neuroinflammation and neurodegeneration by corrupting vital communication pathways between neurons, glial cells, and peripheral immune cells.

### 4.1 Dysregulation of neuroimmune axes in Parkinson’s disease

In the aging brain, essential bidirectional communication between neurons and immune cells becomes significantly disrupted. A central feature of this dysfunction is the senescence-induced alteration of neuron–microglia signaling. Microglia, the resident immune sentinels of the CNS, adopt a primed, pro-inflammatory (M1-like) phenotype, characterized by immunometabolic deficits and sustained release of cytotoxic cytokines. This shift drives chronic neuroinflammation and contributes to the loss of dopaminergic neurons (48). This state can be exacerbated by a phenomenon known as the “kiss of death,” where immune cells like NK cells may acquire inhibitory molecules such as PD-1 through membrane exchange, leading to their functional silencing and reduced ability to clear pathological proteins. This process may disproportionately affect SNpc dopaminergic neurons due to their unique physiological profile.

This neuroinflammatory milieu is further exacerbated by the aberrant reactivation of complement-mediated synaptic pruning. In PD, microglia recognize and engulf synapses tagged with complement proteins such as C1q and C3. Given the extensive axonal arborization of each substantia nigra pars compacta (SNpc) dopaminergic neuron (forming an estimated 0.5–1 million striatal synapses), even a modest increase in complement tagging can lead to widespread synaptic loss. This not only disrupts basal ganglia circuitry but also imposes a significant metabolic burden on the neuronal soma, accelerating neuronal exhaustion.

In Parkinson’s disease (PD), distinct T cell subpopulations play complex and critical roles within the brain parenchyma and perivascular spaces, exhibiting both detrimental and protective functions. CD8+ cytotoxic T cells infiltrate the substantia nigra early in the disease process, even preceding significant alpha-synuclein aggregation and neuronal death (49). These cells, equipped with cytolytic enzymes like granzymes, can directly contact and damage dopaminergic neurons (49). The CD4+ T cell population demonstrates a particular duality. Pro-inflammatory subsets, notably T helper 17 (Th17) cells, are elevated in PD and exacerbate neuroinflammation and neurodegeneration primarily by secreting IL-17A, which activates microglia and amplifies the local inflammatory response (50). Furthermore, a unique subset of cytotoxic CD4+ T cells (CD4 CTLs) has been found to expand in PD patients (51). In contrast, the regulatory T cell (Treg) subset, an anti-inflammatory CD4+ lineage, plays a protective role by suppressing effector T cell and microglial activity, thus helping to maintain immune homeostasis; however, this protective function is often impaired in PD patients (52). The recruitment of these T cells, particularly CD4+ T cells, is significantly influenced by CNS-associated macrophages (CAMs) located at perivascular spaces, which act as key antigen-presenting cells (53). The progressive loss of dopaminergic neurons in PD reduces striatal dopamine, thereby diminishing this immunoregulatory influence and creating a vicious cycle in which neurodegeneration begets neuroinflammation, which in turn accelerates further degeneration (54). The interplay between these infiltrating and resident immune cells ultimately shapes a neuroinflammatory environment that drives the progression of PD.

Conversely, beneficial gut microbes ferment dietary fiber to produce short-chain fatty acids (SCFAs), which exert potent immunomodulatory effects. SCFAs promote the integrity of the gut barrier, support the generation of peripheral Tregs, and can cross the blood–brain barrier (55). Within the CNS, they function as histone deacetylase inhibitors, promoting an anti-inflammatory microglial phenotype and enhancing the clearance of pathological proteins. Thus, an age-related decline in SCFA production may reduce these protective effects, contributing to a CNS environment susceptible to PD pathogenesis.

### 4.2 Abnormal communication between glial cells

**Figure 3:**
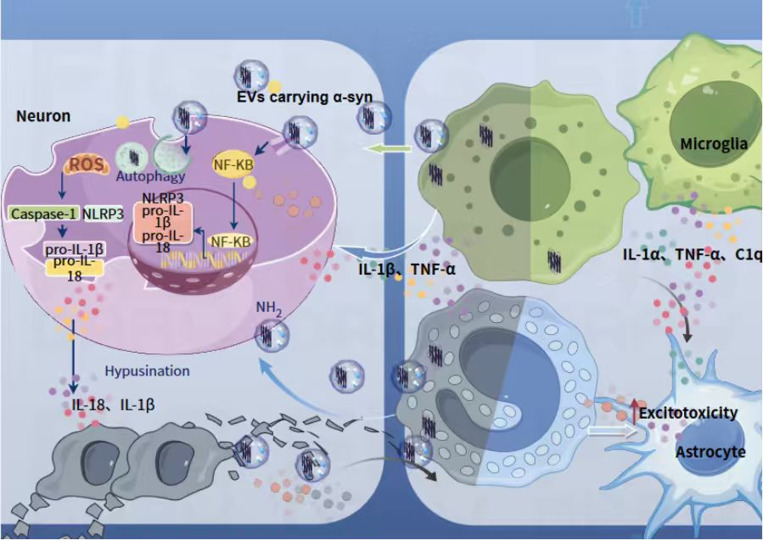
Neuro-immune and glial dysregulation driven by inflammaging in PD. Exosomes mediate transcellular delivery of inflammatory mediators and aggregated α-synuclein to recipient cells. Activated microglia secrete proinflammatory cytokines to polarize astrocytes into the neurotoxic A1 phenotype, and drive the formation of a persistent neuroinflammatory cycle in the brain via activation of the NLRP3 inflammasome and related signaling pathways. Senescent neuroglial cells display an intrinsic proinflammatory phenotype and release abundant cytotoxic cytokines. Immunosenescence impairs the physiological crosstalk between neurons and neuroglial cells, thereby inducing a chronic neuroinflammatory microenvironment in the brain and ultimately causing degenerative damage to midbrain dopaminergic neurons.

The cross-talk between microglia and astrocytes, fundamental to CNS homeostasis, becomes severely dysregulated in PD. In the context of neuroinflammation, activated microglia release pro-inflammatory cytokines that drive astrocytes into a neurotoxic A1 state (**Figure 3**). These A1 astrocytes subsequently release factors that impair neuronal function and accelerate neurodegeneration (56). Key molecular pathways, particularly the NLRP3 inflammasome, sustain this cycle, promoting chronic inflammation and neuronal death (57). Aging exacerbates these alterations, as senescent glial cells exhibit impaired communication and heightened inflammatory responses (58).

As presented in **Figure 3**, This maladaptive interaction creates a self-perpetuating cycle via autocrine and paracrine signaling, where astrocytes sustain microglial activation and vice versa. Aging also compromises the supportive functions of glial cells: aged astrocytes show reduced capacity for glutamate uptake, promoting excitotoxicity, while aged microglia shift toward a neurotoxic phenotype, releasing damaging factors instead of neuroprotective molecules (59–61). The convergence of aging, glial dysfunction, and chronic neuroinflammation creates a vulnerable CNS environment substantially susceptible to PD pathology.

Promising strategies to target dysregulated glial cross-talk include inhibiting the NLRP3 inflammasome to break the activation cycle, converting A1 astrocytes to protective A2 phenotypes, using senolytics to clear senescent glial cells, neutralizing specific cytokines with monoclonal antibodies, and restoring astrocytic metabolic support and glutamate clearance capacity. Future work should prioritize delineating the specific signaling pathways underlying pathological glial interactions through human post-mortem studies and advanced *in vitro* models, while developing CNS-penetrant therapeutics that target multiple aspects of this dysregulated communication. Clinical translation will require biomarker strategies to identify patients with prominent glial activation and assess target engagement. Combination approaches that address both inflammatory signaling and age-related functional decline in glia represent a promising avenue for achieving meaningful disease modification in PD.

### 4.3 Exosome-mediated intercellular communication

Exosomes (30–150 nm extracellular vesicles) serve as critical mediators of intercellular communication, playing a dual role in both propagating pathology and offering therapeutic opportunities. During aging and neurodegeneration, senescent microglia and astrocytes release exosomes loaded with inflammatory mediators (e.g., IL-1β, TNF-α) and pathological proteins, particularly aggregated α-synucleins (62, 63). The packaging of misfolded α-synuclein within exosomes is a key mechanism for its intercellular spread, with exosome-associated aggregates showing greater neurotoxicity than their free forms (64, 65). This role in pathogenesis underscores the potential of exosomal cargo composition as a diagnostic biomarker, reflecting the pathophysiological state of donor cells.

Beyond their pathological role, exosomes hold significant promise as therapeutic vectors. Their innate ability to cross the blood-brain barrier (BBB) makes them attractive for targeted drug delivery. Engineered exosomes can be loaded with therapeutic cargo, such as siRNA or neuroprotective factors, for precise delivery to affected neurons (66, 67). Recent research has significantly advanced our understanding of exosome-mediated disease mechanisms, revealing a complex landscape beyond simple cargo transfer. It is now evident that exosomes from a single cell type are not uniform; they comprise distinct subpopulations with varying membrane lipid compositions (e.g., cholesterol and phospholipid content), as identified by techniques like Laser Tweezers Raman Spectroscopy, suggesting cells may produce specific exosome “subtypes” for different communicative tasks (68). Furthermore, while miRNAs remain important, the cargo diversity now includes other functional nucleic acids like long non-coding RNAs (lncRNAs) and circular RNAs (circRNAs), which are highly stable and can promote processes such as metastasis or malignant transformation, offering new diagnostic and therapeutic avenues. Crucially, the mechanism behind their cell-specific targeting, or “homing,” is better elucidated, involving interactions between surface proteins (e.g., tetraspanins, integrins) and receptors on recipient cells, which explains patterns like organotropic metastasis and is vital for designing targeted therapies (69).

In summary, while your original text correctly outlines the fundamental dual nature of exosomes, the latest research has expanded into manipulating their inherent heterogeneity, engineering them with unprecedented precision for targeted drug delivery, and applying them in innovative clinical settings from hair restoration to neurodegenerative diseases, bringing the promise of exosome therapy closer to reality.

### 4.4 Interaction between immunosenescence and genetic risk factors for PD

The pathogenic link between immunosenescence and PD is further elucidated through dysregulated epigenetic mechanisms. Age-related alterations in DNA methylation significantly influence transcriptional activity and immune cell homeostasis. For instance, hypermethylation of immune regulatory genes can suppress protective responses, while genome-wide hypomethylation may facilitate the derepression of pro-inflammatory mediators, collectively accelerating neurodegeneration (70). Furthermore, histone modifications and non-coding RNAs (e.g., miRNAs) fine-tune inflammatory cascades by targeting key signaling nodes, offering potential as both biomarkers and therapeutic targets.

Complementing these changes, histone post-translational modifications-such as acetylation of H3K9 and H3K27-enhance chromatin accessibility and promote pro-inflammatory cytokine production in microglia. Aberrant histone modifications sustain a hyperinflammatory microglial phenotype, contributing to dopaminergic neuron loss, whereas histone deacetylase inhibitors (HDACs) demonstrate therapeutic potential by restoring microglial balance. Furthermore, non-coding RNAs (e.g., miR-124, miR-155, lncRNAs like NEAT1) fine-tune inflammatory cascades by targeting key signaling nodes (e.g., NF-κB/STAT pathways) and modulating neuroprotective versus neurodegenerative transcriptional programs. Integrated profiling of these regulatory networks reveals disease-specific signatures, offering dual utility as biomarkers and therapeutic targets (70). The interplay between DNA methylation, histone modifications, and non-coding RNAs deciphers the mechanistic complexity of immunosenescence in PD, providing a framework for epigenetic reprogramming strategies aimed at delaying neurological decline (71).

## 5 Potential targets for personalized medical strategies for PD

**Figure 4:**
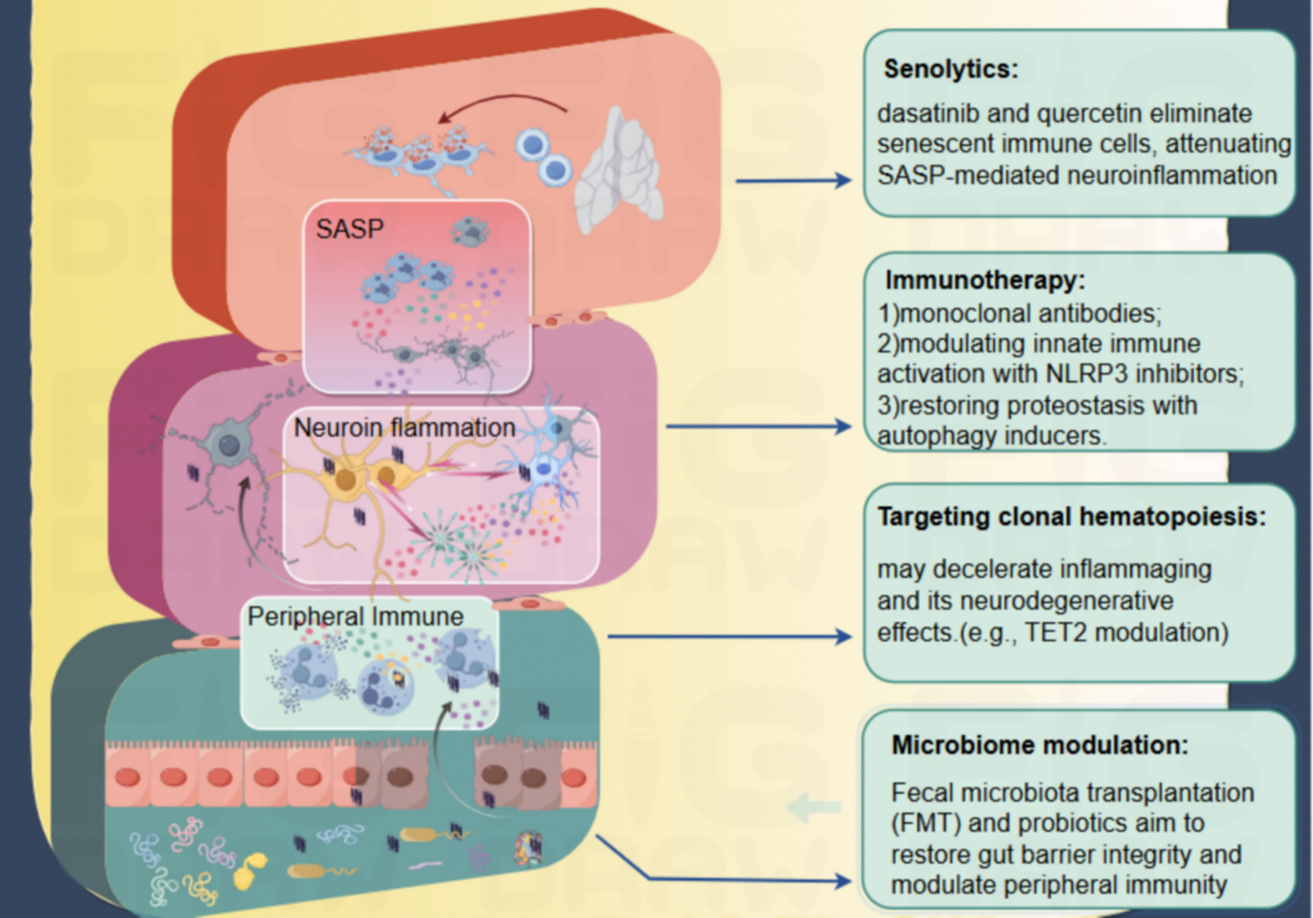
Therapeutic strategies targeting immunosenescence and inflammaging in PD. 1) Hallmarks of immunosenescence (e.g., thymic involution, accumulation of senescent immune cells) impair immunosurveillance and reduce the clearance of pathological α-syn. This defect activates microglia, triggers neuroinflammation, and disrupted protein homeostasis further exacerbates abnormal α-syn accumulation. 2) Intrinsic microglial senescence induces SAPs, characterized by a hyperactivated SASP, impaired phagocytosis, and persistent neuroinflammation—all of which increase the vulnerability of dopaminergic neurons. 3) Peripheral immunosenescence disrupts neuro-immune crosstalk: chronic inflammation, BBB dysfunction, and gut microbiota dysbiosis synergistically promote the infiltration of peripheral immune cells into the CNS, further activating glial cells and perpetuating α-syn-related pathology. Potential therapies targeting peripheral immunosenescence include senolytics, immunotherapeutic interventions, microbiome modulation, and targeted therapy for CHIP.

Personalized therapeutic strategies for PD are increasingly focused on integrating genetic, epigenetic, and biomarker-driven approaches. As shown in **Figure 4**, Genetically informed immune modulation leverages pathogenic variants in genes such as GBA, LRRK2, and APOE, which dysregulate neuroinflammatory pathways and accelerate neurodegeneration (72–74). Genetic risk stratification enables the design of precision immunotherapies that recalibrate immune activity in a patient-specific manner, improving efficacy and reducing off-target effects. Furthermore, genetic risk stratification helps identify actionable biomarkers that predict treatment response, supporting the optimization of individualized therapeutic algorithms (72–74). Complementarily, epigenome-targeted therapies aim to restore physiological gene expression without altering DNA sequences. Agents such as HDAC inhibitors (e.g., vorinostat) and DNA methyltransferase modulators (e.g., 5-azacytidine) have shown promise in attenuating neurodegeneration in preclinical models, though challenges remain in achieving blood-brain barrier penetration and mitigating off-target transcriptional effects (75, 76).

Biomarker-driven optimizationis critical for personalized management; clinically validated indicators like neurofilament light chain (NfL) and glial fibrillary acidic protein (GFAP) provide objective measures of disease progression and therapeutic response. Real-time biomarker profiling facilitates dynamic treatment adjustments, supporting precision neurology frameworks (77, 78). Ultimately, advancing personalized medicine requires integrating genetic background, epigenetic modifications, and biomarker-guided strategies to develop tailored interventions that address individual disease biology.

### 5.1 Targeted immunaging strategies

Non-steroidal anti-inflammatory drugs (NSAIDs) have been investigated as potential disease-modifying agents in PD, primarily targeting neuroinflammation, a well-established component of its pathogenesis. This interest is supported by epidemiological studies suggesting a reduced risk of PD development among users of certain NSAIDs, with a more pronounced effect observed for non-aspirin NSAIDs like ibuprofen compared to aspirin (79). However, clinical trials evaluating the efficacy of NSAIDs in slowing disease progression or alleviating motor symptoms in patients with established PD have yielded inconsistent and largely neutral results (80). The therapeutic prospects of long-term NSAID use are further tempered by their significant adverse effect profile, which includes gastrointestinal, renal, and cardiovascular risks, necessitating careful patient selection and vigilant monitoring. Consequently, the current evidence regarding the therapeutic benefit of NSAIDs in manifest PD remains equivocal. While preclinical mechanistic studies robustly support their anti-inflammatory and neuroprotective properties in models, clinical translation has been hampered by heterogeneous trial designs, inadequate sample sizes, and insufficient attention to disease stage-specific effects. The absence of biomarker-guided patient stratification has further limited the interpretability of existing clinical investigations (81).

Future research requires larger, well-designed clinical trials to definitively ascertain the potential role of NSAIDs in PD management and to identify specific patient subgroups that may derive benefit.

### 5.2 Cytokine-targeted immunomodulation

Targeting these inflammatory pathways presents a promising avenue for developing disease-modifying therapies in PD. Preclinical evidence demonstrates that targeted inhibition e.g., IL-1 receptor antagonism attenuates microglial activation and reduces neuronal damage, suggesting genuine disease-modifying potential (82). Monoclonal antibodies against specific cytokines like IL-1β or TNF-α offer a precise approach. Preclinical studies demonstrate that neutralizing these cytokines can attenuate neuroinflammation and protect dopaminergic neurons (83, 84). Additionally, the risk of disrupting physiological immune responses requires careful evaluation of long-term immunological consequences (85). Small-molecule inhibitors targeting key inflammatory signaling nodes show significant promise. For instance, JAK/STAT pathway inhibitors (e.g., AZD1480) and NLRP3 inflammasome inhibitors have been shown to suppress microglial activation and mitigate neurodegeneration in PD models (86). Natural compounds like baicalein, a flavonoid, have also demonstrated efficacy in attenuating neuroinflammation by inhibiting the NLRP3/caspase-1/GSDMD pathway (87).

Future research should prioritize several key interconnected areas to advance neuroinflammation-targeted therapies. A primary focus is the development of novel CNS-penetrant compounds or sophisticated delivery systems designed to effectively cross the blood-brain barrier (BBB), thereby ensuring therapeutic agents reach their intended targets within the central nervous system. Furthermore, exploring rational combination therapies that simultaneously target multiple nodes of the neuroinflammatory network—such as inhibiting a specific cytokine (e.g., IL-1β via NLRP3 inflammasome targeting) (**Figure 4**) while enhancing regulatory T-cell function—holds promise for achieving synergistic therapeutic effects and overcoming the limitations of single-target approaches (88, 89). The discovery and validation of reliable biomarkers detectable in cerebrospinal fluid (CSF) or through advanced neuroimaging techniques (e.g., TSPO PET) are equally crucial for effective patient stratification, monitoring target engagement, and assessing treatment response in clinical trials. Ultimately, advancingpersonalized medicine approaches is key; this involves tailoring interventions based on an individual’s genetic background (e.g., APOE ϵ4 or TREM2 variant status), immune profile, and specific disease stage to significantly improve therapeutic outcomes.

In conclusion, while harnessing neuroinflammation as a therapeutic target in PD presents significant challenges, the growing understanding of its central role in disease progression continues to drive the development of innovative and promising immunomodulatory strategies. Future success will likely depend on overcoming translational barriers and adopting a more personalized treatment approach.

### 5.3 Regulatory T cell Treg therapy

Adoptive regulatory T cell (Treg) therapy represents an emerging immunomodulatory strategy for mitigating chronic neuroinflammation in PD. Tregs, characterized by the CD4+CD25+FOXP3+ phenotype, are essential for maintaining immune homeostasis through multifaceted suppressive mechanisms, including cell-contact-dependent inhibition, cytokine sequestration, and modulation of antigen-presenting cells. Preclinical PD models demonstrate that enhancing Treg function or abundance attenuates microglial activation, reduces pro-inflammatory cytokine release, and promotes dopaminergic neuron survival (82). Therapeutic approaches encompass ex vivo expansion of autologous Tregs followed by adoptive transfer, or pharmacological induction of *in vivo* Treg differentiation using low-dose IL-2 or mTOR inhibitors (90). However, translational challenges persist, including the identification of disease-specific Treg subsets, functional instability in inflammatory microenvironments, and risks of unintended immunosuppression (91). Ongoing research aims to optimize Treg-based protocols by leveraging antigen-specific chimeric antigen receptor (CAR)-Tregs and combinatorial regimens to achieve sustained neuroprotection and disease modification in PD (92).

Recent years have witnessed significant breakthroughs in Treg therapy research for PD patients. A core finding is that Tregs in PD patients are not only reduced in number but also exhibit impaired immunosuppressive function (93). However, *in vitro* expansion technology has successfully reversed this functional impairment: Treg cells obtained from PD patients, after expansion, not only increase in number but also see their immunosuppressive capacity restored and even enhanced (93). These expanded Treg cells show upregulated expression of key functional markers (such as FOXP3, CD25, CD73, CTLA-4, and IL-10) and regain the crucial ability to suppress pro-inflammatory myeloid cells, which is lacking in untreated Treg cells from patients. To further enhance treatment precision and efficacy, the research field is moving from polyclonal Treg therapy towards antigen-specific therapy (94). One cutting-edge strategy involves developing Treg cells specific for alpha-synuclein (α-syn), a core pathological protein in PD. Preclinical studies indicate that, compared to polyclonal Treg cells, these α-syn-specific Treg cells can migrate more efficiently to α-syn-enriched regions in the brain, thereby providing stronger neuroprotective effects. These include more effectively ameliorating motor deficits, reducing the loss of dopaminergic neurons, inhibiting α-syn accumulation, and modulating microglial activation (94).

These advancements are paving a multifaceted path toward clinical application, utilizing non-invasive stimulation, immunologic drug repurposing, and sophisticated engineered cell products to accelerate the translation of Treg therapy for PD.

### 5.4 Senolytic strategies for PD

Senolytic agents-compounds that selectively eliminate senescent cells-constitute a transformative therapeutic paradigm for PD by targeting cellular senescence, a key driver of neuroinflammation and neurodegeneration (**Figure 4**). Currently, the most widely studied senescent cell clearance agents mainly include dasatinib combined with quercetin and navitoclax (ABT263). Their pharmacokinetic characteristics, particularly the brain exposure concentration, are crucial for evaluating their potential in treating neurodegenerative diseases. Preclinical studies demonstrate that senolytics such as dasatinib and quercetin reduce senescent cell burden in PD models, attenuating glial activation, NLRP3 inflammasome signaling, and α-synuclein pathology (95). These agents act via inhibition of apoptosis-resistance pathways (e.g., BCL-2, PI3K/AKT), thereby clearing senescent microglia and astrocytes while ameliorating motor and cognitive deficits (40, 96). Beyond cellular clearance, senolytics exert pleiotropic neuroprotective effects through antioxidant and anti-inflammatory mechanisms, preserving mitochondrial function and synaptic integrity (97). Nonetheless, clinical translation faces significant hurdles: long-term safety profiles in humans remain undefined, and off-target effects (e.g., thrombocytopenia with BCL-2 inhibitors) necessitate rigorous risk-benefit evaluation (91).

In PFF-injected mice, clearance of senescent glia using ABT-263 (navitoclax) prevented neuronal loss and motor decline (98). Innovative approaches, such as chiral gold nanoparticles functionalized with antibodies targeting senescent cell markers (e.g., B2MG, DCR2), have also been shown to selectively clear senescent microglia under light exposure, alleviating PD-like pathology in mice (99). Studies have shown that dasatinib can partially penetrate the blood-brain barrier after oral administration. A preliminary clinical study on patients with mild Alzheimer’s disease revealed that after oral administration of dasatinib and quercetin, dasatinib could be detected in the blood and cerebrospinal fluid of the patients. Specifically, in the blood of all 5 participants who completed the 12-week treatment, dasatinib was detected (concentration range 12.7–73.5 ng/ml), and in the cerebrospinal fluid of 4 participants (80%), a lower concentration of dasatinib (range 0.281–0.536 ng/ml) could also be detected, with a cerebrospinal fluid/blood plasma concentration ratio of approximately 0.422–0.919%. However, in the same study, although quercetin was detected in the blood (3.29–26.3 ng/ml), it did not reach a detectable level in the cerebrospinal fluid (100). This indicates that dasatinib can enter the central nervous system, but its concentration is relatively low, while the central penetration of quercetin may be very limited.

On the other hand, preclinical studies have provided more promising data for another senescent cell clearance agent, navitoclax. A study conducted in elderly non-human primates (crucial monkeys) found that after intermittent oral administration of navitoclax, a drug concentration sufficient to clear senescent cells could be achieved in the cerebrospinal fluid, and positive signals were observed, such as a reduction in senescence-related biomarkers (such as SASP factors) in the brain and an improvement trend in the activation of microglia (evaluated through TSPO-PET imaging) (101). This proves that navitoclax can effectively enter the central nervous system in higher mammals and may exert biological effects. Future efforts must prioritize CNS-penetrant senolytic formulations, biomarker-driven patient stratification, and combinatorial approaches integrating immunomodulation to maximize therapeutic efficacy.

The latest advances in senolytic strategies for PD integrate markedly targeted nanotechnology, molecular pathway modulation, and innovative immunotherapy combinations to address core pathological mechanisms. For instance, chiral gold nanoparticles functionalized with anti-B2MG and anti-DCR2 antibodies can selectively bind to senescent microglia and, upon near-infrared light exposure, activate the Fas signaling pathway to induce apoptosis-effectively clearing toxic cells without harming healthy ones, which in PD mouse models reduced α-synuclein levels in cerebrospinal fluid by approximately 90% and improved motor function (99). Concurrently, modulating the SUMOylation pathway-such as by inhibiting the deSUMOylation enzyme SENP1 or overexpressing the SUMO conjugase Ubc9-has been shown to stabilize α-synuclein, mitigate oxidative stress, and enhance dopaminergic neuron survival in preclinical models, offering a complementary molecular approach to suppress protein aggregation and cellular stress (102).

In summary, senotherapy aims to modify PD progression by eliminating a key driver of inflammation and neuronal dysfunction—senescent cells. Its potential as a disease- modifying strategy is supported by growing evidence from laboratory models, though clinical validation in humans is still underway.

### 5.5 Targeted delivery systems

#### 5.5.1 CNS-specific nanoplatforms

The development of advanced nanoscale delivery platforms represents a pivotal advancement in senolytic therapeutics for central nervous system disorders. Nanotechnology-based approaches enable precise targeting of senescent cells within the CNS while minimizing systemic exposure and off-target effects. For example, chiral gold nanoparticles functionalized with glutathione demonstrate enantioselective properties, allowing them to selectively induce apoptosis in senescent microglia without damaging healthy neural cells, thereby significantly enhancing therapeutic specificity (99). These innovative systems not only improve the therapeutic index by enabling higher precision with reduced dosing frequency but also facilitate personalized treatment strategies through integration with real-time imaging modalities for dynamic monitoring and optimization (103). Such advancements are aligned with ongoing efforts to develop second-generation senolytics that exhibit improved tissue specificity and enhanced safety profiles.

#### 5.5.2 Critical challenges impede clinical implementation

The translation of senolytic therapies into clinical practice faces four interconnected challenges: the lack of specific biomarkers (such as p16INK4a and SA-β-gal) that can reliably and uniquely identify senescent cells across different tissues and conditions (46, 104). The substantial heterogeneity among senescent cells, where distinct subtypes (e.g., G2-phase-arrested cells with elevated IL-6 secretion) exhibit unique molecular profiles and varying drug sensitivities; the biological complexity stemming from the dual roles of senescent cells, which can drive pathological processes while also being essential for beneficial processes like tissue repair and wound healing-a complexity underscored by clinical setbacks with non-selective clearance strategies (105) and the persistent difficulty in achieving adequate CNS penetration while maintaining targeting specificity, particularly for multi-pathway combination regimens.

### 5.6 Vaccination-based immunomodulatory strategies

Vaccination-based immunomodulation represents an emerging therapeutic paradigm for Parkinson’s disease, focusing on harnessing the adaptive immune system to target core pathological processes, rather than traditional infectious agents. The primary objective of this strategy is to induce a protective immune response against pathological proteins, most notably alpha-synuclein (a-syn) (106, 107), which aggregates to form Lewy bodies, a hallmark of PD.

The rationale behind this approach is twofold. First, it aims to promote the clearance of pathogenic a-syn species before they can trigger neuroinflammation and neuronal damage. This is achieved by generating antibodies that can opsonize extracellular a-syn, facilitating its removal by microglia. Second, a carefully calibrated immune response can shift the balance from a pro-inflammatory, cytotoxic T-cell response towards a more regulatory or anti-inflammatory phenotype. The core challenge of immunotherapy lies in precisely distinguishing and eliminating toxic α-syn aggregates (such as oligomers and fibrils) while not affecting the physiological α-syn monomers. Different regions (domains) of the α-syn protein may trigger different immune responses. For instance, antibodies targeting the C-terminal (such as Prasinezumab) and those targeting the N-terminal (such as Cinpanemab) show different results in clinical trials (108). The key lies in finding the “target points” (i.e., antigenic epitopes) that are only exposed to the pathogenic aggregates but not to the monomers. However, the various pathological forms and conformational dynamic changes of α-syn make it extremely difficult to screen out universal epitopes that can broadly neutralize toxicity (109). Inappropriate epitope selection may prevent the therapy from effectively binding to the key pathogenic aggregates, which is one of the reasons why some clinical trials did not reach the primary endpoint.

However, the development of these vaccines faces numerous challenges. One of the major difficulties is to achieve a sufficiently specific immune response that targets only the pathological, misfolded form of α-synuclein, without triggering an autoimmune reaction against normal physiological proteins, as this reaction is crucial for normal synaptic function. Additionally, ensuring that the antibodies or immune cells induced by the vaccine can effectively reach the central nervous system is also quite challenging, as there is the blood-brain barrier (BBB). It prevents most macromolecular drugs (such as antibodies) from entering the central nervous system. This results in less than 1% of therapeutic antibodies reaching the brain (110). The inflammatory environment in the brains of Parkinson’s disease patients may increase the permeability of the blood-brain barrier, but this could be a double-edged sword, allowing both beneficial and harmful immune components to enter. To address this issue, researchers have developed “brain shuttle” technologies, such as attaching fragments that can specifically bind to receptors on the blood-brain barrier to antibodies, allowing them to “take a ride” into the brain. A dual-specific antibody called SAR446159 employs this strategy and significantly increased the drug concentration in the brain in preclinical models (110). Nevertheless, how to achieve efficient, safe, and controllable delivery remains a section that needs continuous optimization in drug development.

Current research explores various platforms, including peptide-based vaccines using short, modified sequences of a-syn designed to elicit a targeted antibody response, and DNA/RNA-based vaccines that instruct the body to produce a-syn antigens, leading to a broader immune response. The ultimate aim is to develop a vaccination strategy that can be administered in the pre-symptomatic or early stages of PD to modify the disease course by fundamentally altering the immune system’s interaction with the underlying pathology.

## 6 Immune system rejuvenation strategies

### 6.1 Hematopoietic stem cell transplantation

HSCT represents a clinically established therapeutic strategy, notably effective in certain autoimmune and degenerative conditions, through the infusion of pluripotent stem cells capable of reconstituting diverse hematopoietic lineages. This procedure may contribute to the restoration of age-compromised immune homeostasis by regenerating immune cell populations. Available evidence suggests that HSCT can modulate neuroinflammatory processes, promote neuroprotection, and mitigate chronic inflammation-associated neurodegeneration (111). Importantly, studies indicate that HSCT has the potential to induce durable immune reconstitution (111, 112), which could translate into improved clinical outcomes in neurological disorders in PD. However, the application of HSCT specifically in PD remains investigational, necessitating further rigorous evaluation of its safety and efficacy profiles in this context.

### 6.2 Young plasma-derived factor therapy

Complementing cellular therapeutic strategies, young plasma is enriched with bioactive factors that demonstrate the capacity to counteract age-associated immune dysfunction. Specific components, such as growth factors and cytokines, contribute to enhanced immune competence and exhibit neuroprotective properties. Research has identified several key plasma proteins capable of modulating inflammatory cascades and promoting neuronal integrity (113). Preclinical studies provide evidence that administration of young plasma improves cognitive performance and attenuates neuroinflammation in models of PD (114). While these mechanisms hold substantial promise, translating these findings into clinical applications requires further systematic investigation to establish long-term efficacy and elucidate precise molecular pathways.

### 6.3 Immunometabolic reprogramming

A third therapeutic strategy focuses on metabolic pathways that regulate immune cell function during aging. Immune metabolic reprogramming redirects immunometabolic processes from pro-inflammatory toward anti-inflammatory states, thereby attenuating chronic neuroinflammation associated with PD (115). Key interventions include: 1) caloric restriction and specific dietary modifications that enhance immunometabolic plasticity; and 2) pharmacological agents targeting critical metabolic regulators. These approaches improve immune cell resilience and functionality, potentially delaying PD progression through restoration of anti-inflammatory capacity (116). Collectively, immune rejuvenation strategies-including HSCT, young plasma-derived factor administration, and immunometabolic reprogramming-constitute an emerging conceptual framework for addressing immune dysfunction in PD (**Table 1**). Although these approaches remain largely in preclinical stages, they represent promising avenues for enhancing immune response and controlling neuroinflammation. Prioritizing mechanistic studies and translational research will be essential to establish their potential as disease-modifying immunotherapies for PDs.

**Table 1:** Immunomodulatory strategies for targeting immunosenescence in PD.

Strategy	Mechanism of action	Current stage	Key challenges
Hematopoietic Stem Cell Transplantation (HSCT)	Reconstitutes immune cell populations via pluripotent stem cells; modulates neuroinflammation and promotes neuroprotection	Investigational (preclinical/early clinical)	Safety, efficacy, and durability of immune reconstitution in PD models
Young Plasma-Derived Factor Therapy	Utilizes bioactive factors (e.g., growth factors/cytokines) to counteract age-related immune dysfunction and reduce neuroinflammation	Preclinical	Long-term efficacy, molecular pathways, and clinical translation
Immunometabolic Reprogramming	Redirects immunometabolism from pro- to anti-inflammatory states via dietary/pharmacological interventions (e.g., caloric restriction)	Preclinical	Optimization of metabolic targets and individual variability in response
Targeting Immunosenescence	Inhibits age-related immune decline (e.g., cytokine-driven neuronal damage) through checkpoint inhibitors or lifestyle interventions	Early research	Validation in aging populations and integration with PD pathology

## 7 Current challenges and future directions

### 7.1 Translational challenges and research bottlenecks in animal models

PD research extensively utilizes animal models to investigate disease mechanisms and evaluate therapeutic interventions. However, substantial translational challenges persist due to pathophysiological disparities between these models and human PD (111). Rodent models recapitulate core PD features, such as neuroinflammation and dopaminergic neuron degeneration, yet they fail to fully emulate the multifactorial complexity of human neurodegeneration, which involves intricate gene-environment interactions (117). Key translational bottlenecks in PD research include: 1) oversimplification of the nonlinear progression and comorbidities of human PD in existing models; 2) genetic and epigenetic homogeneity that does not reflect clinical heterogeneity in disease progression and treatment responses; and 3) interspecies divergences in neuroinflammatory mechanisms—while microglial activation is conserved, differential signaling pathways (e.g., NLRP3 dynamics) and cytokine profiles (e.g., IL-1β/TGF-β ratios) contribute to distinct neurodegenerative trajectories (118).

These limitations highlight the imperative for next-generation models that incorporate clinically relevant variables, such as chronological aging, sex-specific pathophysiology, polygenic risk architectures, and environmental exposures. Advanced model systems are expected to enhance the predictive validity of preclinical PD research and facilitate the development of targeted immunotherapeutic strategies (**Table 2**).

**Table 2:** Future research directions for targeting immunosenescence in PD.

Research domain	Specific goals & strategies	Key approaches & technologies
1. Decoding Immunosenescence & Immune Heterogeneity	Elucidate the precise role of immunosenescence in PD pathogenesis and its interaction with neurodegeneration. Identify specific immune cell subsets and their functional states that contribute to neuroinflammation and disease progression. Discover novel biomarkers for early diagnosis, prognosis, and monitoring of therapeutic response.	High-resolution single-cell technologies (e.g., scRNA-seq, mass cytometry) on PBMCs and CNS-derived immune cells. Longitudinal studies to track immune dynamics from pre-symptomatic to advanced stages. Multi-omics integration (transcriptomics, proteomics, epigenomics) of immune cells. Development of immune-specific biomarker panels (e.g., based on CD8+ T cell clusters, NK cell cytotoxicity profiles, monocyte activation states).
2. Advanced Model Systems	Develop next-generation models that better recapitulate the complexity and heterogeneity of human PD. Incorporate clinically relevant variables to improve translational predictive validity. Create human-relevant platforms for pathophysiological study and therapeutic screening.	Organ-on-a-chip models (e.g., neuroimmune chips, BBB-on-a-chip) to study neuro-immune crosstalk, immune cell transmigration, and cytokine-mediated neuronal dysfunction with high spatiotemporal resolution. Patient-derived cell models (iPSCs) for personalized medicine applications. Genetically engineered models incorporating polygenic risk architectures. Models incorporating chronological aging, sex-specific pathophysiology, and environmental exposures (e.g., toxins, gut microbiota).
3. AI-Driven Personalized Medicine & Therapeutics	Develop predictive models for individual disease trajectories and treatment outcomes. Optimize therapeutic intervention timing (therapeutic windows). Accelerate discovery and design of novel, targeted therapeutics.	AI and machine learning algorithms to analyze multi-omics, clinical, and neuroimaging data for early prediction of disease progression. In silico clinical trials using neural network-based simulations of individual pathophysiology to test treatment responses and optimize regimens (e.g., medication dosing). Generative adversarial networks (GANs) for *de novo*design of novel neuroprotective or anti-inflammatory compounds with optimized efficacy and safety profiles.
4. Targeted Immunomodulation & Neuroprotection	Translate identified immune targets into effective disease-modifying therapies. Develop strategies to slow or halt disease progression by modulating neuroinflammation. Overcome the challenge of drug delivery across the blood-brain barrier.	Targeted biologics and small molecules against specific immune pathways (e.g., NLRP3 inflammasome inhibitors, Caspase-1 inhibitors, TLR2/TLR4 modulators, cytokine-specific antibodies). Cell-based therapies (e.g., HSPC transplantation for immune reconstitution). Advanced drug delivery systems: Nanoparticles, viral vectors, and exosomes for targeted CNS delivery; BBB-on-chip platforms for screening permeability.
5. Biomarker Discovery & Validation	Discover and validate sensitive and specific biomarkers for early diagnosis, accurate patient stratification, and objective monitoring of disease progression and treatment response.	Leveraging multi-omics to identify biomarker signatures in blood, CSF, and other peripheral tissues. Advanced neuroimaging techniques(e.g., specific PET ligands for neuroinflammation, α-synuclein aggregates) to visualize pathological changes. Large-scale multicohort verification and methodological standardization for clinical translation.
6. Intersection of Gut-Brain Axis & Immunity	Investigate the role of peripheral immune activation (e.g., in the gut) in initiating or driving CNS pathology. Explore the mechanistic link between microbial dysbiosis, immune function, and PD.	Preclinical and clinical studies exploring gut microbiome modulation (e.g., pre/probiotics, fecal microbiota transplantation) on neuroinflammation. Investigating how gut-derived immune signals (e.g., from activated enteric TLRs) influence central neuroinflammation and α-syn pathology propagation.
7. Focus on Sex-Specific Differences & Hormonal Regulation	Understand the mechanistic basis of sexual dimorphism in PD incidence, progression, and immune responses. Develop sex-stratified therapeutic strategies.	Multi-omics approaches (e.g., single-cell transcriptomics, epigenomics) to delineate how sex hormones (estrogen, androgens) interface with immune aging and α-synuclein pathology. Preclinical studies in models incorporating sexual dimorphism to evaluate sex-specific treatment efficacy.

### 7.2 Immunosenescence pathogenesis in PD

Immunosenescence is considered a key factor in PD, but research in this area faces several interconnected challenges.

#### 7.2.1 Mechanistic and heterogeneity challenges

A primary challenge is unraveling the causal relationship between immunosenescence and PD pathology. It remains difficult to determine if the observed immune changes are a driving force of the disease or a consequence of neuronal degeneration (119). Furthermore, the interaction between the central nervous system (e.g., activated microglia) and the peripheral immune system (e.g., T cells) is greatly complex. The search results indicate that EOPD and LOPD patients exhibit different immune profiles, suggesting distinct underlying mechanisms. For instance, EOPD patients show a shift towards a more pro-inflammatory state in the adaptive immune system (120). This heterogeneity complicates the search for universal therapeutic targets.

#### 7.2.2 Research methodology challenges

Progress is hampered by a shortage of non-invasive biomarkers capable of dynamically assessing the state of immune senescence (e.g., in senescent microglia) in the living brain (119). Additionally, commonly used animal models cannot fully replicate the decades-long process of human immune aging and its interaction with PD pathology, limiting the translatability of preclinical findings.

#### 7.2.3 Therapeutic development challenges

Translating immunosenescence research into safe and effective treatments faces significant hurdles. A major difficulty is precise immunomodulation. Immune cells like microglia can have both protective and harmful roles; simply suppressing or activating the entire immune system is not viable. The goal is to finely rebalance the immune system, which is exceptionally challenging (121). Another hurdle is intervention timing. Immune system dysregulation may begin years or even decades before motor symptoms appear. While intervening at a pre-symptomatic stage is ideal, identifying at-risk individuals and conducting such interventions pose substantial clinical and ethical challenges.

In summary, the main challenges in studying the central role of immunosenescence in PD lie in deciphering its complex and causal mechanisms, overcoming methodological limitations in research, and developing targeted therapies that can safely and effectively modulate the immune system to alter the course of the disease.

### 7.3 Sexual dimorphism in PD

PD demonstrates significant sexual dimorphism in its epidemiological distribution and clinical manifestations, with males exhibiting a higher incidence than females (approximately 1.5–2:1 ratio). Emerging research indicates that these disparities are modulated by sex-specific variations in immunosenescence, involving age-related immune dysfunction and chronic inflammation (122). Hormonal factors, particularly estrogen, confer neuroprotective effects in females by regulating immune responses and suppressing neuroinflammation. Estrogen activates α-estrogen receptor (ERα) signaling, which inhibits pro-inflammatory microglial polarization and reduces the release of cytokines such as TNF-α and IL-1β, while concurrently preserving mitochondrial integrity in dopaminergic neurons (111). Conversely, the decline in estrogen levels during menopause heightens susceptibility to neurodegenerative processes. Additionally, sex-based differences in immune reactivity-including divergent microglial activation thresholds, phagocytic capacity, and cytokine production contribute to disparate clinical outcomes. CX3CR1^+^ pro-inflammatory microglial infiltration is more pronounced in male patients, whereas females retain stronger anti-inflammatory repair capabilities, potentially linked to X-chromosome dosage effects and androgen/estrogen balance (118). Elucidating these mechanisms is crucial for developing sex-stratified therapies tailored to distinct immunological profiles. Future studies should integrate multi-omics approaches (e.g., single-cell transcriptomics, epigenomics) to delineate how sex hormones interface with immune aging and α-synuclein pathology, thereby enabling precision medicine paradigms for PD.

Current research on the sexual dimorphism in PD faces several interconnected challenges that complicate the translation of these findings into effective, sex-stratified therapies.

#### 7.3.1 Mechanistic complexity of hormonal actions

A primary challenge is the complex and often contradictory role of sex hormones, particularly estrogen. While epidemiological data suggests a neuroprotective effect, evidenced by increased PD risk in women who undergo early bilateral oophorectomy, the clinical picture is nuanced. Hormonal effects are sexually dimorphic; for instance, in experimental models, estradiol promotes adaptive responses in females but can have negligible or even suppressive effects on striatal dopamine in males. Furthermore, the distinction between the effects of peripheral (gonadal) hormones and those produced locally in the brain adds another layer of complexity, as they may have different impacts on neuronal survival and inflammation (123). This makes it difficult to predict the outcome of hormone-based therapies.

#### 7.3.2 Navigating therapy development

Translating these mechanistic insights into safe and effective treatments presents significant hurdles. A major difficulty is designing precise hormonal interventions that account for the timing, specific compound, and dosage. Evidence suggests that the neuroprotective effect of estrogen in animal models is most consistent when administered before a neurotoxic insult, highlighting the critical importance of intervention timing. The use of Selective Estrogen Receptor Modulators (SERMs) like tamoxifen is a promising approach, but its effects can be double-edged, as it has been associated with both neuroprotection in models and an increased risk of PD in clinical cohorts (124). This underscores the challenge of achieving beneficial effects without triggering adverse outcomes.

#### 7.3.3 Methodological and diagnostic hurdles

Progress is hampered by several methodological limitations. A significant issue is the lack of reliable biomarkers to dynamically track immune aging and sex-specific pathological processes in patients (125). Additionally, commonly used animal models have limitations in fully replicating the complex, long-term interaction between human immune aging, hormonal fluctuations, and PD pathology (123). There is also a need to better integrate the understanding of how sex hormones interface with other key disease mechanisms, such as alpha-synuclein pathology and mitochondrial dysfunction, which remains unclear.

### 7.4 Challenges in translational medicine

Translational medicine encounters three principal barriers in developing novel therapies for PD:

#### 7.4.1 Biomarker development & validation

Biomarker development is constrained by pathophysiological complexity that limits specificity and sensitivity, exemplified by spatiotemporal heterogeneity in neuroinflammatory and neurodegenerative processes such as those in PD. Furthermore, analytical validation requires large-scale multicohort verification, while methodological standardization remains challenging due to the absence of unified discovery protocols (126).

#### 7.4.2 Optimization of therapeutic windows

The presence of diagnostic delays-where pathological changes precede clinical symptoms by years-complicates the optimization of therapeutic windows. Dynamic disease progression, influenced by factors such as immunosenescence, exhibits significant interindividual variability. This necessitates precision medicine approaches that integrate biomarker profiling, disease staging, and multi-omics stratification to tailor intervention timing.

#### 7.4.3 Advanced drug delivery systems

The blood-brain barrier (BBB) excludes >98% of neurotherapeutics, posing dual challenges: the biological complexity of carrier-tissue interactions requiring comprehensive pharmacokinetic and pharmacodynamic characterization, and translational infrastructure deficits that demand scalable manufacturing and regulatory-compliant clinical trial designs for novel delivery systems (117). Addressing these challenges requires integrated strategies: leveraging multi-omics to spatially and temporally map neurodegenerative processes; deploying AI-driven models to predict individualized therapeutic windows based on biomarker trajectories; and advancing BBB-on-chip systems coupled with *in vivo* imaging for quantifying drug biodistribution. Implementing these approaches through academic-industrial collaboration is essential to bridge the gap between preclinical discovery and clinical application in neurodegenerative therapeutics.

### 7.5 Future research directions

#### 7.5.1 Decoding immunosenescence

Recent advances in single-cell technologies have accelerated research on immunosenescence in neurodegenerative diseases, particularly PD (PD), by enabling high-resolution characterization of immune heterogeneity and its role in neuropathology. Single-cell RNA sequencing facilitates the deciphering of transcriptional profiles of immune cells during aging and neuroinflammation, revealing immunosenescence-mediated dysregulation associated with PD progression. This approach identifies dysfunctional immune subsets and aberrant activation states in aging individuals, with alterations that correlate closely with disease severity (118). Furthermore, the resolution of immune heterogeneity at the single-cell level holds potential for yielding novel biomarkers for early PD diagnosis and prognosis, as well as identifying therapeutic targets (122). Future research should prioritize the integration of single-cell analyses with longitudinal assessments to track immune dynamics across disease progression and interventions, thereby elucidating mechanistic links between immunosenescent remodeling and clinical outcomes.

#### 7.5.2 Organ-on-a-chip models for advancing neuro-immune research

Organ-on-a-chip platforms, building upon single-cell methodologies, embody a transformative advancement for neuroimmunology research. These microphysiological systems recapitulate intricate neuroimmune crosstalk within precisely regulated microenvironments, thereby enabling pathophysiologically relevant modeling of neuroinflammatory and neurodegenerative processes (127, 128). Representative blood-brain barrier-on-a-chip (BBB-on-a-chip) models elucidate the dynamics of immune cell transmigration and cytokine-mediated neuronal dysfunction with high spatiotemporal resolution (129). This integrated approach significantly accelerates therapeutic discovery by permitting real-time evaluation of compounds targeting neuroinflammatory pathways. Furthermore, by clarifying cell-specific mechanistic drivers of neurodegeneration in human-relevant contexts, these platforms promote the identification of precision therapeutics (130). Future investigations should prioritize: 1) enhancing physiological fidelity through advanced multicellular compartmentalization and dynamic perfusion parameters; and 2) pursuing robust clinical validation using patient-derived cells to facilitate personalized medicine applications for Parkinson’s disease and related disorders.

#### 7.5.3 AI-driven personalization in neurodegenerative disease management

Artificial intelligence (AI) is fundamentally reshaping personalized therapeutics for PD by leveraging integrated multi-omics, clinical, and neuroimaging data to enable data-driven precision medicine (131). Through deep learning models that analyze longitudinal biomarkers, AI facilitates the early prediction of disease trajectories and enables proactive intervention (132). Furthermore, neural network-based simulations of individual pathophysiology allow for the in silico testing of treatment responses, optimizing therapeutic regimens such as medication dosing or neuromodulation parameters. In drug discovery, generative adversarial networks (GANs) are being employed to design novel compounds with optimized efficacy and safety profiles. To translate these advances into clinical practice-particularly in PD-key steps include prospective validation in multi-center cohorts, alignment of regulatory standards (e.g., FDA and EMA), and real-world trials to verify generalizability and patient benefit.

## 8 Conclusions

This review has synthesized compelling evidence establishing immunosenescence as a critical pathogenic nexus in Parkinson’s disease (PD). The interplay between systemic immune aging, characterized by inflammaging and SASP, and central nervous system-specific inflammation creates a self-perpetuating cycle that drives neuroinflammation, compromises neuronal homeostasis, and accelerates dopaminergic degeneration. Key mechanisms include microglial senescence, aberrant neuro-immune communication, BBB dysfunction, and SASP-mediated propagation of α-synuclein pathology. These processes are further modulated by genetic risk variants, epigenetic remodeling, and peripheral contributions from the gut-brain axis.

Therapeutic strategies aimed at reversing or mitigating immunosenescence-such as senolytics, Treg-based therapies, cytokine-targeted agents, and immune rejuvenation approaches—hold significant promise for modifying PD progression. However, clinical translation remains challenged by biological complexity, patient heterogeneity, and limitations in preclinical models. Future progress will depend on leveraging single-cell technologies for patient stratification, developing CNS-penetrant therapeutics, validating biomarkers for targeted interventions, and employing human-relevant model systems like organ-on-a-chip platforms. Ultimately, integrating mechanism-based immunomodulation with personalized medicine frameworks represents a pivotal strategy for developing disease-modifying therapies capable of disrupting the vicious cycle of immunosenescence and neurodegeneration in PD.
